# Chicago Medical Society

**Published:** 1887-03

**Authors:** 


### CHICAGO MEDICAL SOCIETY.

Officially Reported for The Atlanta Medical and Surgical Journal.

Stated Meeting, January 3, 1887.

The President, Edmund J. Doering, M. D., in the chair.

Dr. E. J. Kuh read a paper on

### THE ETIOLOGY AND CURE OF ASTHMA, of which the following is a brief synopsis:

The work of Wilhelm Hack on the radical treatment of migraine, asthma, hay fever and other neuroses has received very inadequate recognition in this country. By writers on hay fever he is frequently quoted in an off-hand manner, together with a string of other authors, so that one derives the impression that few of those who quote him have read him; and if his specialistic colleagues do not do him justice, the large class of general practitioners ignore him almost altogether. His work is, taken altogether, of even greater interest to the physician than to the specialist, and it is a deplorable consequence of specialistic exclusiveness that the results of his work have not yet received wider recognition among us. He teaches us that the rhinoscope must forthwith be as indispensable an instrument for all physicians as the thermometer and stethoscope.

The value of Hack's discovery, that asthma nervosum is a reflex disease with usually the nose as a starting point, can best be appreciated by one who himself for many years struggled against the disease and fumed at the utter impotence of medical art to stave off the attacks. If I, therefore, in the course of this paper, class myself among my own patients, I shall do so with the view of bringing the subject within closer range. It is foreign to my subject to consider the isolated publications, from Voltolini downward, on the dependence of asthma upon polypous growths in the nose. Such cases are infrequent enough to be almost considered curiosities (Michel, for instance, reports 135

cases of polypus without asthma); and, as Hack shows, polypi have rather a tendency to prevent asthma than to cause it. It will also simplify our subject if we omit hay fever from our consideration.

The form of asthma of which I wish to treat exclusively is that perennial form which is more or less independent of the seasons, namely, asthma nervosum, or "essentielles asthma" of the Germans. Some persons never get beyond a slight hint of asthma. They will from time to time make a heaving, sighing motion, or complain of præcordial fullness with or without palpitation, or of sudden drowsiness, or dream heavily at night and complain of dullness, lassitude and headache in the morning. This latter condition has many gradations, the culmination of which is nightmare. In the future we must therefore learn to distinguish between an incubus of gastric and of respiratory origin. Other half-asthmatics complain only of a fleeting, leaden heaviness in the limbs amounting almost to pain, the same sensation of which so many true asthmatics complain after an asthmatic night. The typical asthma nervosum is known to us all as a neurosis occurring in paroxysms.

The patient may or may not feel an aura. He will generally, towards evening, or when he lies down, or awakes in the night, begin to wheeze. This wheezing may be associated with itching in the nose, or sneezing, or coughing; the attacks last an indefinite time, and generally end with the expectoration of a transparent glassy mucus. Such patients are often free from asthma during the day. Physical and chemical irritants, such as dust, sudden changes in temperature, the inhalation of certain gases, and a long series of idiosyncrasies which we find enumerated in text-books, can induce an attack. But the recumbent position is the most uniform exciting cause of the single paroxysms. Such patients may be free from chronic bronchitis, chronic emphysema, heart, kidney, intestinal and uterine disease; hence the term "essentielles asthma."

A true etiology of asthma had therefore to be discovered, and Hack did it in the following manner: He knew, of course, of the occasional rôle of nasal neophlasm. Schäffer and B. Fränkel had also indicated that the sensibility of the nasal lining could be so heightened through chronic catarrhal conditions as to be a starting point for reflex disturbances. Then Hack found that he could experimentally produce glottis-spasm by touching the turbinated bodies of a sensitive individual with a probe. He then reasoned as follows: A nasal mucous membrane which shows merely slight affection, and which is not deadened in its sensibility by thickening and hypertrophy, is perhaps a better surface for exciting reflexes than one which shows evident signs of disease; and if this were the case, he reasoned, then perhaps the importance of nasal reflexes had been formerly overlooked just because of the insignificant abnormities of such a sensitive nose.

The theory of Hack is a simple one, and, although it does not cover all the ground, is a very satisfactory one. He says that the turbinated bodies become engorged through various irritants, and that this vaso-dilatatory disturbance is transmitted to the bronchial tubes in asthma. The turbinated bodies act as accumulators for reflexes, store them up, as it were, and then transmit them to other parts. A destruction of the nasal swelling removes the reflexes. The experiences of numerous writers since 1883 corroborate the correctness of Hack's discovery.

What is it that causes nasal occlusion? I have observed myself so closely in this regard, and have so many corroborative observations of intelligent patients that I can make these positive statements:

Firstly, the fullness of the turbinated bodies is regularly influenced by gravitation and corresponds to the position of the head.

It is further influenced by the temperature, and probably much more so by artificial warmth than cold; an overheated room will almost invariably cause swelling in such patients. But the most dangerous and permanent cause of nasal obstruction is the inhalation of dust.

The time is, I hope, not far distant when our views on the etiology of respiratory diseases will undergo a radical change. The superstition of catching cold has lived too long. The light which micological research has thrown on the etiology of most infective diseases must soon influence us toward a conviction that respiratory diseases are *inhaled*, not caught, and that suppuration in the respiratory tract is as impossible without the presence of micro-organism as it is on a wound. The superstition of "catching cold" is so pernicious because it diverts attention from the entrance-way of disease-generators. It is as impossible to contract an acute bronchitis through temperature influences alone as it is to contract tuberculosis through a cold.

It is therefore of the utmost importance to warn asthmatics that as perfect an avoidance of dust inhalation as is possible in our contaminated surroundings is necessary to prevent a recurrence of their trouble. Not only the dust in the streets, but also that in our houses, is to be avoided as much as possible. Carpets and curtains are great receptacles of dust, and a strict regulation of street sprinkling will, in the course of years, when the true etiology of respiratory diseases will have been recognized, be considered as important a municipal regulation as the regulation of sewerage.

When are we to operate on asthmatics? The more recent the asthmatic trouble and the more pronounced the nasal symptoms the better the prognosis. When complicated with chronic bronchitis and chronic emphysema, the outlook is generally bad. A most thorough examination of heart, lungs, kidneys and intestines should precede any operative interference. In cases of cardiac and nephritic asthma with nasal complications, I have *never* cauterized: Firstly, because it has seemed to me irrational; and, secondly, because I feel so much gratitude towards Hack's discovery that I shun any risk which might discredit it.

In some cases it is very difficult to decide whether an operation should be performed or not. For instance, in cases of long standing, say fifteen or twenty years, in which in the first years the nasal symptoms were very pronounced, but in later years have almost or entirely disappeared, in such cases cauterization is sometimes successful, but generally it is unsuccessful.

Cases in which the asthma is more or less constant and has lost its paroxysmal nature give a doubtful prognosis. It has been a matter of experience with me, that those patients to whom the inhalation of Kidder's pastilles, or the application of cocaine to the nose (four per cent. solution on cotton), gives relief, afford a much better prognosis than others.

In asthmatics in which coughing precedes the attack and all nasal symptoms are missing, nasal cauterization will cure if the cough is a so-called nasal cough.

There are a number of asthmatics, fortunately a minority, who seemingly offer a good prognosis, but with whom, for unknown reasons, the operation will fail. There can now be no doubt that there are other starting points for reflexes in the respiratory tract besides the nose. The works of Trautmann and Tornwaldt have already added the vault of the pharynx to this list.

We must accuse the nose *per exclusionem.* Examine every patient thoroughly in every direction, and examine the nose *last*, is what I should like to advise.

About the operation itself little is to be said. It is, as far as we know, absolutely harmless. I have performed many hundred cauterizations without any noteworthy complications. I have never had any traumatic infection. I insufflate iodoform or iodol upon the wound, introduce a pledget of cotton for a few days, and keep my instruments aseptic.

The results are, on the whole, extremely gratifying. Asthma of many years' standing is sometimes broken after the very first cauterization. Almost all patients are relieved and many cured in the strict sense of the word. Some have relapses which additional cauterization will remove. Others again may relapse with a new reflex sensitiveness in other parts.

Dr. J. A. Robinson, in opening the discussion, said: The facts, which are indeed facts, that have been related in this paper are of interest, not only to the specialist, but to the general practitioner. It has been a fact long known to specialists that obstruction of the passage of air through the nares will give rise to asthma and a great number of articles have been written on this subject. It has also been demonstrated that when operations have been performed that cleared away these obstructions the relief from the asthmatic attacks was complete. This can be easily demonstrated by any physician. Cases of nasal polypus are quite frequent and they do not always fall under the care of a specialist. The operation is generally a very simple one; almost any physician, without special training, can remove nasal polypi, and it is really wonderful to find how many cases of asthma are thus cured. As to asthma being due to other causes, I have no doubt of the truth of the observation made by the author—that is that transitory swelling which takes place in the turbinated bodies in cases of mild irritation. I presume we have all noticed that when we are affected with any acute coryza and go to bed at night, the narium of the side on which we lie becomes obstructed, and if we turn over the other side will become obstructed. This is undoubtedly due to the force of gravitation in a great many cases where the mucous membrane is especially sensitive. There is no doubt that in a great many cases by the irritation of a probe, or the inhalation of dust, coughing can be produced resulting in asthmatic attacks. Therefore, this demonstrates that reflex irritation of the nares is one of the causes of asthma, and it points out very clearly the method of treatment which should be instituted. The author has rendered a service in showing that there are such a large number of cases in which by destroying the turbinated bodies we can prevent the occurrence of reflex asthma. It would have been an interesting question to solve whether, in the case of the author's personal experience, a respirator worn over the nose so that the air could not pass through the nose unless filtered, would have been of any benefit in preventing the recurrence of the asthma.

Dr. H. Martyn Scudder: About five years ago, when practicing in India, where I had to ride on horseback a great deal in the sun and breathe a great deal of dust, I suffered frequently from acute attacks of coryza, accompanied occasionally by bronchitis and slight asthma. The nose was not much obstructed, and when an attack of coryza came on fifteen minutes' sleep would often cause it to pass away. Gradually the attacks became more severe and were accompanied and followed by some obstruction. When in London more than three years ago, Dr. Mackenzie wanted to cauterize my nose, but it was before the days of cocaine and I decidedly objected, as I thought the remedy worse than the disease. Since coming to Chicago I have been troubled less than when abroad. Quite recently I had my nose cauterized by Dr. E. Fletcher Ingals, and it has certainly relieved the trouble to a very great extent. My experience, however, was somewhat different from Dr. Kuh's as the cauterization gave me considerable trouble for a week or two. It was followed by soreness and even by slight chills, and it made me feel out of sorts for about a fortnight, but it was successful in relieving the obstruction, and I have had no more asthma or bronchitis, although once in a while I still suffer from attacks of coryza.

Dr. Josef Zeisler said: Professor Schnitzler, of Vienna, has published a number of cases in which decidedly polypus of the nose has caused asthma, and where by the use of the polypus the asthma was cured. I can confirm what Dr. Kuh has said in regard to the effectiveness of the galvano-cautery. I had a case of a boy 12 years old, who had nearly all his lifetime had chronic eczema of the hands and asthma. Believing that the asthma was in causal relation to the eczema, I referred the patient to Dr. Kuh for treatment of the former trouble, while I prescribed local applications for the hands. Very soon both affections were cured and have remained so for the last year.

Dr. H. N. Moyer asked what the author means by the term essential asthma, whether he means reflex asthma or something different?

Dr. Kuh, in closing the discussion, said: By essential asthma I, of course, mean, as I have been attempting to explain all the evening, reflex asthma—the same asthma which text-books classify as idiopathic, or nervous, or essential asthma. In lieu of these clouded expressions we have now, fortunately, a term by which we express an etiological meaning, namely, nasal asthma. It teaches us again that the term neurosis always smacks of the hypothetical, and that when we speak of any pathological condition as a neurosis, we do so in order to cover ignorance. An asthmatic individual is not necessarily a "nervous" one, although I, of course, am not blind to the fact that some unknown factor must come into play in order to affect disease through nasal reflex. In regard to Dr. Zeisler's remarks on the connection between polypi and asthma, I did not know that Schnitzler, of Vienna, had published forty cases of nasal polypus with asthma. I am greatly surprised that such a publication should have escaped my notice. I quoted Michel, of Berlin, as having reported 135 cases of polypus without asthma. There is no doubt that sometimes nasal polypi cause asthma, but, as far as I am aware, only exceptionally so. Hack found that when a patient had polypus with asthma and he left the polypus untouched and cauterized only the turbinated bodies, the asthma disappeared, although the polypi remained in the nose. I think there can be no better evidence of the relative innocence of polypi than that experiment. I have been asked whether, if I had worn a respirator, I would have been free from asthma in traveling. I found that to be the case, for when I plugged my nose with cotton while traveling, I remained free from asthma. Dr. Scudder said that nasal cauterization gave him trouble for weeks. This could only have been through wound complication. An asthmatic may have very severe trouble for a week or less after cauterization on account of the eschar. In order to show how careful one must be in diagnosis, I should like to interpolate the following description: A patient with the mildest form of asthma, namely, the occasional involuntary deep, sighing inspiration, consulted me. The examination was negative with the exception of slight tympanites (the abdomen should always be carefully examined in such cases) and slight swelling of the inferior turbinated bodies. I treated his mild constipation for weeks without any benefit to his respiratory trouble. Then I cauterized, also without effect. At last I discovered that his alæ nasi were so pliable that when he inhaled through the nose they collapsed and occluded the nares. In regard to the claim that injections of boracic acid solution into the nose will relieve asthma, I should simply refer to the uniformly condemnatory verdict of all specialistic practitioners against the use of the nasal douché in such cases.

